# COVID-19 and Acute Macular Neuroretinopathy – An underlying association?

**DOI:** 10.1016/j.amsu.2022.103847

**Published:** 2022-05-26

**Authors:** Warda Ahmed, Azeema Suri, Aymen Ahmed

**Affiliations:** a Medical College, The Aga Khan University, Karachi, 74800, Pakistan; b Department of Medicine, Dow University of Health Sciences, Karachi, Pakistan

Acute Macular Neuroretinopathy (AMN) is a rare condition of unknown etiology marked by a wedge-shaped reddish-brown lesion reaching towards the fovea and resulting in a rapid onset of one or more paracentral scotomas [1] The reddish “flower petal” lesions associated with reduced visual acuity [2], appear three days to two months after the first onset of symptoms and may persist for an indefinite period. Some, however, partially resolve on their own. AMN is diagnosed by multimodal imaging and is commonly seen in young females. It has been associated with various risk factors including flu and caffeine, the most common being oral contraceptive pills. Given reports of a surge in the incidence of AMN during the COVID-19 pandemic [3], it is necessary to speculate upon the association of COVID-19 and AMN.

A study conducted in a hospital at France found that while eleven cases of AMN were identified during the SARS-CoV 2 pandemic in 2020, only one had been reported the year before, that is, 2019 [4]. Of these eleven patients, four (36%) underwent testing for COVID-19 and had a positive polymerase chain reaction (PCR) test. The association between the two diseases is further emphasized in a case report by *Preti RC* et al., where AMN was detected as the first sign of COVID-19 infection in a patient who later developed fever and cough while testing positive for COVID-19 [5]. A similar case was reported by *Masjedi M* et al.*,* where a 29-year-old woman developed AMN two weeks following a positive COVID-19 test [1]. Likewise, another case of simultaneous occurrence of AMN and COVID-19 was reported by *Aidar MN* et al. [6].

Moreover, an interesting correlation between AMN and Adenovirus-based COVID-19 vaccines has been lately observed. *Franchi A* et al. reported two cases of young females presenting with AMN within 48 h of administration of the COVID-19 Oxford AstraZeneca vaccine, a non-replicating viral vector vaccine [7]. Similarly, *Gabrielle* et al. reported a case of bilateral AMN in a 25-year-old woman 24 h following administration of the first dose of the same vaccine [8]. Additionally, *Valenzuela* et al. reported a case of AMN following administration of the Pfizer-BioNTech COVID-19 vaccine [9]. Similar findings have also been reported in several other case reports [[10], [11], [12]]and may be suggestive of a causal relationship between COVID-19 vaccination and AMN that is yet to be established.

Viral illnesses like flu and vaccines have already been associated with AMN. Although not precise, the underlying mechanism has been hypothesized to be a vascular or thromboembolic event as a result of the inflammatory reaction, leading to ischemia of the deep retinal capillary plexus and the formation of scotomas [3]. SARS-CoV-2 enters host cells via interaction of its spike protein with an ACE-II receptor, a receptor interestingly found in the retinal ganglion cell layer, inner plexiform layer, inner nuclear layer, and photoreceptor outer segments as well. Additionally, the TMPRSS2, necessary for the virus's action, is found in multiple retinal neuronal cells, vascular and perivascular cells, and in retinal Müller glial cells [13]. Coupled with this, COVID-19 has demonstrated to induce an autonomous dysregulation of blood flow to the choroid, thereby causing deprivation of oxygen and nutrients and consequent hyperoxygenation of the outer retina, thereby making the retina more prone to oxidative damage. Therefore, these mechanisms could possibly moderate the pathophysiology of AMN in COVID-19 patients.

In conclusion, COVID-19 is often characterized as a respiratory illness due to its much common involvement of the respiratory tract. However, it has been reported to lead to complications involving other organs as well. Here, we have highlighted its possible involvement in retinal disorders and loss of visual acuity. Further research needs to be done to confirm the association of AMN with COVID-19 and its vaccines. Clinical presentation of the cases being presented can be used to investigate the different pathogenic mechanisms of COVID-19, which can help us better understand this novel association [4]. This can also provide more clarity on the etiopathogenesis of AMN itself.

## Ethical approval

Since this paper did not involve patients, no ethical approval was required.

## Sources of funding

No funding was acquired for this paper.

## Declaration of competing interest

The authors declare that there is no conflict of interest.

## Consent

This study was not done on patients or volunteers, therefore, no written consent was required.

## Author contribution

Warda Ahmed: conception of the study, major contribution towards drafting the manuscript, and agreeing to the accuracy of the work. Azeema Suri: conception of the study, major contribution towards drafting the manuscript, and agreeing to the accuracy of the work. Aymen Ahmed: conception of the study, major contribution towards drafting the manuscript, and agreeing to the accuracy of the work.

## Guarantor

The Guarantor is the one or more people who accept full responsibility for the work and/or the conduct of the study, had access to the data, and controlled the decision to publish.

Warda Ahmed, Azeema Suri, Aymen Ahmed.
